# Xprediction: Explainable EGFR-TKIs response prediction based on drug sensitivity specific gene networks

**DOI:** 10.1371/journal.pone.0261630

**Published:** 2022-05-18

**Authors:** Heewon Park, Rui Yamaguchi, Seiya Imoto, Satoru Miyano

**Affiliations:** 1 M&D Data Science Center, Tokyo Medical and Dental University, Bunkyo-ku, Tokyo, Japan; 2 Division of Cancer Systems Biology, Aichi Cancer Center Research Institute, Chikusa-ku, Nagoya, Aichi, Japan; 3 Division of Cancer Informatics, Nagoya University Graduate School of Medicine, Showa-ku, Nagoya, Aichi, Japan; 4 Human Genome Center, The Institute of Medical Science, The University of Tokyo, Minato-ku, Tokyo, Japan; Osmania University, Hyderabad, India, INDIA

## Abstract

In recent years, drug sensitivity prediction has garnered a great deal of attention due to the growing interest in precision medicine. Several computational methods have been developed for drug sensitivity prediction and the identification of related markers. However, most previous studies have ignored genetic interaction, although complex diseases (e.g., cancer) involve many genes intricately connected in a molecular network rather than the abnormality of a single gene. To effectively predict drug sensitivity and understand its mechanism, we propose a novel strategy for explainable drug sensitivity prediction based on sample-specific gene regulatory networks, designated Xprediction. Our strategy first estimates sample-specific gene regulatory networks that enable us to identify the molecular interplay underlying varying clinical characteristics of cell lines. We then, predict drug sensitivity based on the estimated sample-specific gene regulatory networks. The predictive models are based on machine learning approaches, i.e., random forest, kernel support vector machine, and deep neural network. Although the machine learning models provide remarkable results for prediction and classification, we cannot understand how the models reach their decisions. In other words, the methods suffer from the black box problem and thus, we cannot identify crucial molecular interactions that involve drug sensitivity-related mechanisms. To address this issue, we propose a method that describes the importance of each molecular interaction for the drug sensitivity prediction result. The proposed method enables us to identify crucial gene-gene interactions and thereby, interpret the prediction results based on the identified markers. To evaluate our strategy, we applied Xprediction to EGFR-TKIs prediction based on drug sensitivity specific gene regulatory networks and identified important molecular interactions for EGFR-TKIs prediction. Our strategy effectively performed drug sensitivity prediction compared with prediction based on the expression levels of genes. We also verified through literature, the EGFR-TKIs-related mechanisms of a majority of the identified markers. We expect our strategy to be a useful tool for predicting tasks and uncovering complex mechanisms related to pharmacological profiles, such as mechanisms of acquired drug resistance or sensitivity of cancer cells.

## Introduction

Drug sensitivity prediction and related markers identification are critical tasks in precision medicine. In recent years, drug response prediction has drawn increasing attention in the various research fields because of growing interest in precision medicine and the availability of pharmacogenomics datasets from large-scale projects (e.g., Genomics of Drug Sensitivity in Cancer, PRISM drug repurposing resource).

Although various computational methods (e.g., machine learning including deep neural network) have been developed for drug response prediction [1–3], molecular interactions have been largely ignored in the previous studies. Heterogeneous genetic networks are crucial to understanding complex mechanisms of diseases (e.g., cancer), because the molecular mechanisms underlying diseases reflect the perturbations in the specific function of molecules in the complex cellular network, rather than a consequence of an abnormality in a single gene [4]. Thus, the molecular interactions underlying a disease are crucial to understand the mechanism related to drug sensitivity of cell lines and can provide vital information for drug response prediction. The effectiveness of the networks-based analysis has been proven in various research works, e.g., cancer prediction, drug combinations identification, and protein-protein interaction [5–7]. However, only a few studies have been devoted to drug sensitivity prediction based on gene networks [8–12]. Kim et al. [10] proposed a prediction method (DrugGCN) based on deep learning approaches. The DrugGCN considered protein-protein interaction (PPI) network and mapped gene expression levels to each gene on PPI network. Then, the constructed graph with expression levels of genes was used as input of convolution network to predict drug responses. Sokolov et al. [11] developed a prediction model based on the network constrained regularization method, called generalized elastic net. The method incorporates gene networks into elastic net penalty, and thus encourages smoothness of the coefficients on features connected in network. Yang et al. [12] proposed a novel strategy to learning multiple tasks (prediction of multiple drugs) and identifying feature interaction based on the Macau. They generated the interaction matrix between feature of drugs and features of cell lines, and predicted drug response based on interaction of protein targets of drug and the pathway’s activity. The existing studies on network-based drug sensitivity prediction were conducted by an averaged molecular network for all samples, i.e., cell line specific characteristics of molecular interaction were ignored. However, the strengths of the relationships between genes vary depending on cell line characteristics, e.g., cancer progression and drug sensitivity, and the dynamic genetic networks reflecting characteristics of cell lines provide crucial information for personalized medicine.

This study proposes a novel strategy for explainable drug sensitivity prediction based on sample-specific gene regulatory networks, called Xprediction. First, we estimate sample-specific gene regulatory networks under varying conditions of samples, i.e., the *n* networks are estimated for *n* cell lines. Then, the estimated multiple networks are used as input features of the model for drug sensitivity prediction. We consider machine learning approaches, i.e., random forest (RF), kernel support vector machine (kSVM), and deep neural network (DNN), for the predictive model. Although the machine learning approaches, including deep neural networks, provide effective prediction accuracy, interpretation of the prediction results by the approaches remains challenging. That is, the machine/deep learning approaches suffer from the black box problem (i.e., the approaches cannot explain why the model reaches a certain conclusion). In the black box model, our concern is the verifying impact of input variables on the outputs and their importance, because the result can be explained by the importance of the input features. To understand the importance of each molecular interaction on the drug sensitivity prediction result, we propose a method that measures the impact of molecular interactions on drug sensitivity prediction. In the proposed method, the importance of molecular interactions is measured by comparison of prediction results obtained with and without the gene-gene interaction.

We apply the proposed Xprediction to predict five EGFR tyrosine kinase inhibitors (TKIs), afatinib, dacomitinib, erlotinib, gefitinib, and osimertinib, where gefitinib and erlotinib are the first, afatinib and dacomitinib are the second, and osimertinib is the third-generation EGFR-TKIs. The five EGFR-TKIs were approved to treat EGFR mutation-positive non-small cell lung cancer (NSCLC) and have a common target gene, EGFR. Erlotinib has an additional target gene, NR1I2, and afatinib is also used for ERBB2 and ERBB4 -targeted treatment. From the practical perspective, our interest lies in determining whether a cell responds sensitively to a drug or not, rather than the sensitivity value of a cell. Thus, we consider the binary classification problem of drug sensitive and resistant cell lines. For 536 cancer cell lines, we first estimate drug sensitivity-specific gene networks under varying EGFR TKI sensitivity of cell lines. We then predict drug sensitivities of other EGFR TKIs not used for gene network estimation. Our strategy based on sample-specific gene regulatory networks shows effective prediction results compared with prediction based on expression levels of genes. We then identified crucial molecular makers for EGFR TKIs prediction. The identified crucial markers show different regulatory systems between drug sensitive and resistant cell lines. The EGFR TKIs related mechanisms of the identified markers are verified through the literature. To the best of our knowledge, this study is the first on explainable drug sensitivity prediction based on sample-specific gene networks.

The remainder of this paper is organized as follows: The Materials and methods section introduces datasets used for drug sensitivity-specific gene regulatory networks estimation and EGFR TKIs prediction. We then introduce a sample-specific gene network estimation method and propose a method for explainable drug sensitivity prediction (Xprediction). We then describe EGFR TKIs prediction results and the identified markers. Conclusions are provided in the Discussion section.

## Materials and methods

### Datasets

Drug sensitivity-specific gene regulatory networks were constructed by gene expression data and drug sensitivity obtained from the Cancer Dependency Map (DepMap) Potal (https://depmap.org/portal/). RNA-expression of 19,144 genes and 1,305 cell lines was obtained from the CCLE dataset (20Q). Drug sensitivity (DS) of 4,686 compounds and 578 cell lines was obtained from the PRISM repurposing primary screen. CRISPR-Cas9 screening describing gene dependency was also downloaded. From 19,144 genes, we extract 1,168 genes that matched with 1,732 candidate regulators (i.e., 1,183 transcription factors, 47 nuclear receptors and 502 miRNA) used in [13], as well as 1,000 genes with the highest variance in expression levels, where 70 genes are duplicated in the two extracted genes sets. We consider the extracted 2,098 genes as candidate regulators and 19,144 genes as targets.

We focused on the five EGFR-TKIs, afatinib, dacomitinib, erlotinib, gefitinib, and osimertinib, and extracted 536 cell lines by matching cell lines of gene expression, CRISPR-Cas9 screening, and the sensitivities of the EGFR-TKIs without missing values. We then constructed 536 afatinib sensitivity-specific gene networks for 536 cell lines. For the other four EGFR-TKIs, all 536 networks were estimated.

### Methods

Assuming $X_{1},\ldots ,X_{p}\in R^{n}$ are the expression levels of *p* = 2, 098 possible regulators that control transcription of the expression levels of *l*^*th*^ target gene $Y_{l}\in R^{n}$, we considered the following varying coefficient model [14] to construct a gene regulatory network of *α*^*th*^ sample,

$$\begin{array}{c} Y_{l}=\sum_{j=1}^{p}\beta_{jl}\left(m_{\alpha }\right)\cdot X_{j}+\varepsilon_{l}, \end{array}$$ (1)

where ***ε***_*l*_ is a random error vector ***ε***_*l*_ = (*ε*_*l*1_, …, *ε*_*ln*_)^*T*^, which is assumed to be independently and identically distributed with mean 0 and variance *σ*^2^, and *β*_*jl*_(*m*_*α*_) is the regression coefficient of *j*^*th*^ regulator gene (*X*_*j*_) on *l*^*th*^ target gene (*Y*_*l*_) for the *α*^*th*^ sample having modulator (i.e., drug sensitivity) value *M* = *m*_*α*_.

The varying coefficients were estimated by the following kernel based L_1_-type regularization method, called NetworkProfiler [13],

$$\begin{array}{c} L\left(\beta_{l\alpha }\right)=\frac{1}{2}\;\sum_{i=1}^{n}{\left\{y_{il}\;-\;\sum_{j=1}^{p}\beta_{jl}\left(m_{\alpha }\right)x_{ij}\;\right\}}^{2}K\left(m_{i}-m_{\alpha }|b_{l}\right)\;+P\left(\beta_{l\alpha }\right), \end{array}$$ (2)

where ***P***(***β***_*lα*_) is the *L*_1_-type penalty (e.g., elastic net [15]) and

$$\begin{array}{c} K\left(m_{i}-m_{\alpha }|b_{l}\right)=\text{exp}\{\frac{-{\left(m_{i}\;-\;m_{\alpha }\right)}^{2}}{b_{l}}\} \end{array}$$ (3)

is the Gaussian kernel function with a bandwidth *b*_*l*_. The kernel function is used to group cell lines according to the drug sensitivity, and it controls the weight of each cell line on the modeling. For each EGFR-TKI, we estimate 536 gene networks consisting of 19,144 target and 2,098 regulator genes (i.e., 536 matrices consisting of 19,144 rows and 2,098 columns) by using NetworkProfiler. Then, we performed drug sensitivity prediction based on the constructed drug sensitivity-specific gene networks.

For each drug sensitivity-specific gene network, we focused on genes that have the highest correlation between drug sensitivity and their CRISPR-Cas screening. In other words, we computed the correlation between CRISPR-Cas screenings of 18,119 genes and drug sensitivity, and extracted each 182 genes that had the top 1% highest correlation for five EGFR-TKIs, respectively. For target genes matched with each 182 genes, we computed the following regulatory effect in the *α*^*th*^ sample as follows [13]:

$$\begin{array}{c} {\text{RE}}_{\alpha lj}={\hat{\beta }}_{lj}\left(m_{\alpha }\right)\cdot x_{\alpha j},\;\text{for}\;j=1,\ldots ,2098, \end{array}$$ (4)

where *x*_*αj*_ is the expression level of *j*^*th*^ regulator on *α*^*th*^ cell line. For the *α*^*th*^ cell line, the regulatory effect matrix $R_{\alpha }={\left({\text{RE}}_{\alpha 11},\ldots ,{\text{RE}}_{\alpha LJ}\right)}^{T}\in R^{L\times J}$ was computed, where *L* is the number of the selected genes and *J* is the number of regulator genes (i.e., *L* = 182, 180, 182, 181, 182 for afatinib, dacomitinib, erlotinib, gefitinib, osimertinib, respectively, and *J* = 2, 098).

We defined drug sensitive and resistant cell lines based on 10^th^ (10P) and 90^th^ (90P) percentiles of PRISM repurposing primary screen, i.e., sensitive cells: DS<P10 and resistant cells: DS>P90, (P10 values are -1.31, -1.36, -1.31, -1.40, -1.30 and P90 values are 1.06, 1.04, 0.98, 0.59, 0.82 for afatinib, dacomitinib, erlotinib, gefitinib and osimertinib, respectively). We then constructed a network-based predictive model based on the following kernel support vector machine (kSVM), random forest (RF), and neural networks (NN).

- Kernel support vector machine (kSVM)
  SVM is the most widely used machine learning approach for regression prediction and classification. SVM finds boundaries correctly classifying the observations by maximize the distance separating the elements of classes. For nonlinear classification tasks, SVM was extended by applying kernel function based on kernel trick, i.e., kernel SVM, as follows
  $$\begin{array}{c} \underset{\delta }{\text{max}}\left\{\sum_{i=1}^{n}\delta_{i}-\frac{1}{2}\sum_{i=1}^{n}\sum_{h=1}^{n}\delta_{i}\delta_{h}DS_{i}DS_{h}G\left(r_{i},r_{h}\right)\right\} \end{array}$$ (5)
  $$\begin{array}{c} \text{subject}\;\text{to}\;\delta_{i}\geq 0\;i=1,\ldots n,\;\sum_{i=1}^{n}\delta_{i}DS_{i}=0, \end{array}$$ (6)
  where *DS*_*i*_ is a class label of *i*^*th*^ cell line and ***r***_*i*_ is reshaped *L* × *J*-dimensional vector of ***R***_*i*_. In our analysis, we used the following Radial Basis Function (Gaussian kernel function),
  $$\begin{array}{c} G\left(r_{i},r_{h}\right)=\text{exp}(-||r_{i}-r_{h}{\left|\right|}^{2}/2\sigma^{2}) \end{array}$$ (7)
  where *σ* defines the width of the kernel.
- Random forest (RF)
  Random forest is an ensemble learning model based on a combination of multiple decision trees that are constructed by bootstrapped training samples. We first generate *B* bootstrapped samples composed of different features. The *B* decision trees are created using each *B* bootstrapped samples. After the multiple trees are generated, the classification results from the trees are voted for the most popular class.
- Deep neural networks (DNN)
  Artificial neural network (ANN) is a machine learning algorithm inspired by neural networks in brain. ANN consists of three types of layers of nodes, i.e., input layer, a hidden layer, and output layer depending on the function. The input layer connects the external input and the values of predictors are transferred to units of hidden layers. The hidden layers are layers of nodes between input and output layers, and the output layer is the final layer that directly outputs prediction results. DNN is an ANN with multiple hidden layers. In our analysis, a six hidden layered, fully-connected feed-forward neural network was used. We used the *ReLU* activation function on the hidden layers, and the Soft-max function was used on the output layer.

In the models, the multiple regulatory effect matrices obtained from a drug (e.g., afatinib) sensitivity-specific gene networks were considered as input features, and sensitivity of other EGFR-TKIs (e.g., dacomitinib, erlotinib, gefitinib and osimertinib) were considered as the response variables.

However, the machine learning approaches suffer from the black box problem, making it difficult for explainable drug sensitivity prediction. To settle on the issue, we propose a method describing the importance of molecular interaction on drug sensitivity prediction. To measure the feature’s impact on the prediction results, we constructed a model by removing a feature (i.e., removing molecular interaction between *l*^*th*^ target and *j*^*th*^ regulator genes) individually and predicting the drug sensitivity of cell lines (${\hat{y}}^{\left(l,j\right)}$). We then defined prediction accuracy Acc $\left({\hat{y}}^{\left(l,j\right)}\right)$ of the model. The impact of each molecular interaction was measured by comparing with prediction accuracy based on all molecular interactions Acc $\left(\hat{y}\right)$ as follows.

Let *N*_*it*_ is a number of iterations for computing prediction accuracy from the randomly constructed cross validation dataset, $\bar{\text{Acc}(\hat{y})}$ and $s_{\hat{y}}$ are mean and standard deviation of the prediction of accuracies in *N*_*it*_ iterations, respectively. In the model without (*l*, *j*) interaction, corresponding notations are $N_{it}^{\left(l,j\right)}$, $\bar{\text{Acc}({\hat{y}}^{\left(l,j\right)})}$ and $s_{{\hat{y}}^{\left(l,j\right)}}$, respectively. We performed the following t-test,

$$\begin{array}{c} T_{lj}=\frac{\bar{\text{Acc}(\hat{y})}-\bar{\text{Acc}({\hat{y}}^{\left(l,j\right)})}}{s_{p}\sqrt{\frac{1}{N_{it}}+\frac{1}{N_{it}^{\left(l,j\right)}}}} \end{array}$$ (8)

where $s_{p}=\sqrt{\frac{s_{\hat{y}}\left(N_{it}-1\right)+s_{{\hat{y}}^{\left(l,j\right)}}\left(N_{it}^{\left(l,j\right)}-1\right)}{N_{it}+N_{it}^{\left(l,j\right)}-2}}$. Then, the feature’s impact (crucialness of (*l*, *j*)^*th*^ interaction: *I*_*lj*_) on the drug sensitivity prediction was measured by the p.value of the t-test. The detail is given as follows,

**Algorithm 1** Xprediction: explainable drug sensitivity prediction

1: The predictive models were constructed by kSVM, RF, and NN.

2: Predict drug sensitivity by *k*-fold cross validation (CV), i.e., the average of the prediction accuracies of *k* validation sets was given as: Acc $\left(\hat{y}\right)$.

3: Step 2 is iterated *N*_*it*_ times for randomly constructed *k*-fold CV datasets.

4: If *l* ≤ *L* then do

5:  If *j* ≤ *J* then do

6:   Delete (*l*, *j*) elements from regulatory effect matrices.

7:   Predict drug sensitivity without (*l*, *j*) elements: Acc $\left({\hat{y}}^{\left(l,j\right)}\right)$.

8:   Steps 7 is iterated $N_{it}^{\left(l,j\right)}$ times for randomly constructed *k*-fold CV datasets.

9: Perform t-test between Acc $\left(\hat{y}\right)$ and Acc $\left({\hat{y}}^{\left(l,j\right)}\right)$ obtained from *N*_*it*_ and $N_{it}^{\left(l,j\right)}$ iterations, and compute p.value.

10: Extract crucial molecular interaction based on p.value.

## Results

In this section, we have described explainable EGFR-TKIs sensitivity prediction results. We predicted the sensitivities of EGFR-TKIs based on the constructed drug sensitivity-specific gene regulatory networks. For example, we considered four predictive models for predicting gefitinib sensitivity, where inputs of the models were the sensitivity specific gene networks of four drugs (i.e., afatinib, dacomitinib, erlotinib, and osimertinib). The predictive model was generated using 10-fold cross-validation. The first 90% of cells were selected as a training set, while the remaining 10% cells were used as a validation set. For each validation set, the following prediction accuracy was measured,

$$\begin{array}{c} Acc_{cv}=\frac{TP+TN}{TP+TN+FP+FN},\;cv=1,..,10, \end{array}$$ (9)

where TP and TN are the numbers of true positive/negative (an outcome where the model correctly predicts the sensitive/resistant cell), and FP and FN are the numbers of false-positive/negative (an outcome where the model incorrectly predicts the sensitive/resistant cell), respectively. We computed the average of the prediction accuracy of 10 validation sets, i.e., $\text{Acc}(\hat{y})=\sum_{cv=1}^{10}Acc_{cv}$, and the process was repeated 50 times. We also performed drug sensitivity prediction based on expression levels of all 19,144 genes and randomly selected *L* = 182, 180, 182, 181, 182 genes for afatinib, dacomitinib, erlotinib, gefitinib, osimertinib, respectively, without consideration of molecular interaction (Expression (all) and Expression (L) in column “Network” of Table 1), and 50 repeated 10-fold cross-validations to compute prediction accuracy.

**Table 1:** Drug sensitivity prediction accuracy based on gene networks.

	Network	Method	Drug				
			afatinib	dacomitinib	erlotinib	gefitinib	osimertinib
Accuracy	afatinib	NN		0.935	0.922	0.885	0.925
		KSVM		0.946	**0.924**	0.887	**0.927**
		RF		**0.948**	0.897	0.853	0.912
	dacomitinib	NN	0.956		0.887	0.872	0.888
		KSVM	**0.962**		0.897	0.888	0.902
		RF	0.951		0.867	0.850	0.890
	erlotinib	NN	0.874	0.918		0.872	0.899
		KSVM	0.897	0.889		0.887	0.872
		RF	0.865	0.864		0.840	0.846
	gefitnib	NN	0.846	0.841	0.812		0.891
		KSVM	0.836	0.849	0.817		0.898
		RF	0.823	0.859	0.807		0.871
	osimertinib	NN	0.901	0.921	0.826	0.886	
		KSVM	0.892	0.923	0.865	**0.906**	
		RF	0.883	0.898	0.809	0.888	
	Expression (all)	NN	0.809	0.721	0.771	0.771	0.761
		KSVM	0.825	0.740	0.761	0.752	0.790
		RF	0.815	0.712	0.759	0.761	0.822
	Expression (L)	NN	0.770	0.767	0.724	0.807	0.765
		KSVM	0.762	0.754	0.757	0.823	0.778
		RF	0.763	0.770	0.719	0.813	0.813
	Expression (Pan)	NN	0.765	0.758	0.720	0.805	0.769
		KSVM	0.759	0.746	0.754	0.824	0.779
		RF	0.758	0.750	0.729	0.813	0.822
F1 score	afatinib	NN	0.888	0.912		0.931	0.918
		KSVM	0.891	**0.914**		**0.943**	**0.922**
		RF	0.850	0.885		0.941	0.908
	dacomitinib	NN	0.860	0.879	0.954		0.882
		KSVM	0.881	0.885	**0.960**		0.894
		RF	0.836	0.863	0.949		0.880
	erlotinib	NN	0.868		0.871	0.911	0.892
		KSVM	0.884		0.890	0.879	0.865
		RF	0.824		0.850	0.844	0.833
	gefitinib	NN		0.801	0.842	0.828	0.882
		KSVM		0.788	0.818	0.829	0.887
		RF		0.780	0.800	0.844	0.857
	osimertinib	NN	0.883	0.806	0.889	0.915	
		KSVM	**0.903**	0.836	0.880	0.913	
		RF	0.882	0.772	0.868	0.883	
	Expression (all)	NN	0.815	0.717	0.768	0.768	0.771
		KSVM	0.822	0.731	0.773	0.773	0.774
		RF	0.813	0.699	0.755	0.755	0.808
	Expression (L)	NN	0.806	0.719	0.778	0.763	0.767
		KSVM	0.823	0.730	0.780	0.760	0.770
		RF	0.811	0.695	0.767	0.766	0.799
	Expression (Pan)	NN	0.767	0.761	0.710	0.806	0.771
		KSVM	0.770	0.754	0.730	0.825	0.773
		RF	0.753	0.751	0.705	0.813	0.811

Furthermore, we consider gene interactions (pan-cancer interactions) existed in all cell lines (1034, 1119, 874, 1373 and 1095 edges are extracted from afatinib, dacomitinib, erlotinib, gefitnib and osimertinib sensitivity specific networks, respectively). Then, we perform EGFR TKIs responses prediction based on the 1,361 genes in the pan-cancer interactions (Expression (Pan) in Table 1). All pan-cancer interactions extracted from five EGFR TKIs sensitivity specific gene networks can be found in the S1 Table.

Table 1 shows the average of the accuracies and F1 score from the 50 iterations, where the column “Drug” indicates predicted sensitivity of the drug and the column “Network” indicates the modulator for drug sensitivity-specific gene networks estimation. As shown in Table 1, network-based prediction provides effective results for drug sensitivity prediction compared with that based on the expression levels of genes. Especially, the afatinib sensitivity-specific gene network showed outstanding performances overall. Among the machine learning approaches, the kSVM provided the best performances for network-based drug sensitivity prediction. The prediction results imply that molecular interaction is crucial to drug sensitivity prediction and may have crucial information to uncover the mechanisms of drug sensitivity of cell lines, which cannot be extracted by expression levels of genes.

### Interpret EGFR-TKIs sensitivity prediction results

To interpret the EGFR-TKIs prediction result, we measured the impact of each gene-gene interaction (i.e., edge) on the prediction results by using Xprediction. We focused on the following best models for the drug sensitivity prediction of each EGFR-TKIs.

- afatinib: kSVM based on dacomitinib sensitivity specific networks
- dacomitinib: Random forest based on afatinib sensitivity specific networks
- erlotinib: kSVM based on afatinib sensitivity specific networks
- gefitinib: kSVM based on osimertinib sensitivity specific networks
- osimertinib: kSVM based on afatinib sensitivity specific networks

**Fig 1:**
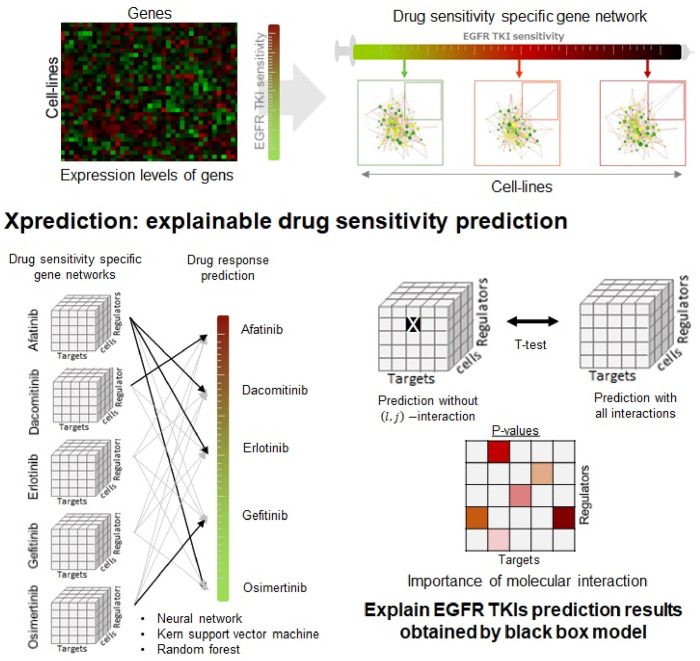
Overall framework of explainable EGFR TKIs prediction.

**Fig 2:**
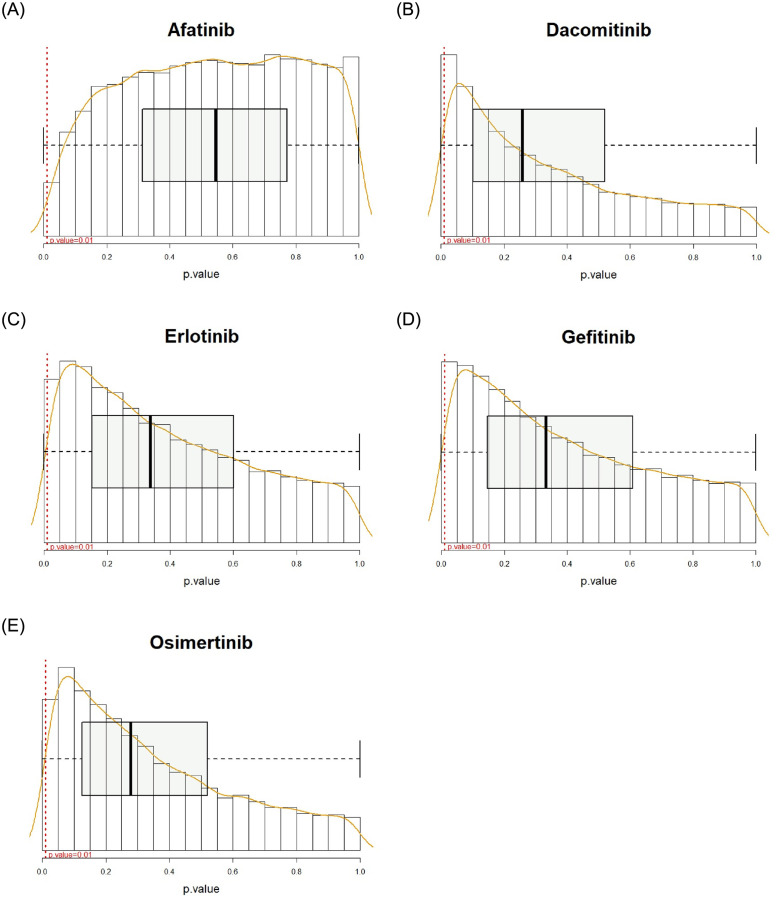
Disribution of importance scores (p-value) of edges for each EGFR-TKIs.

**Fig 3:**
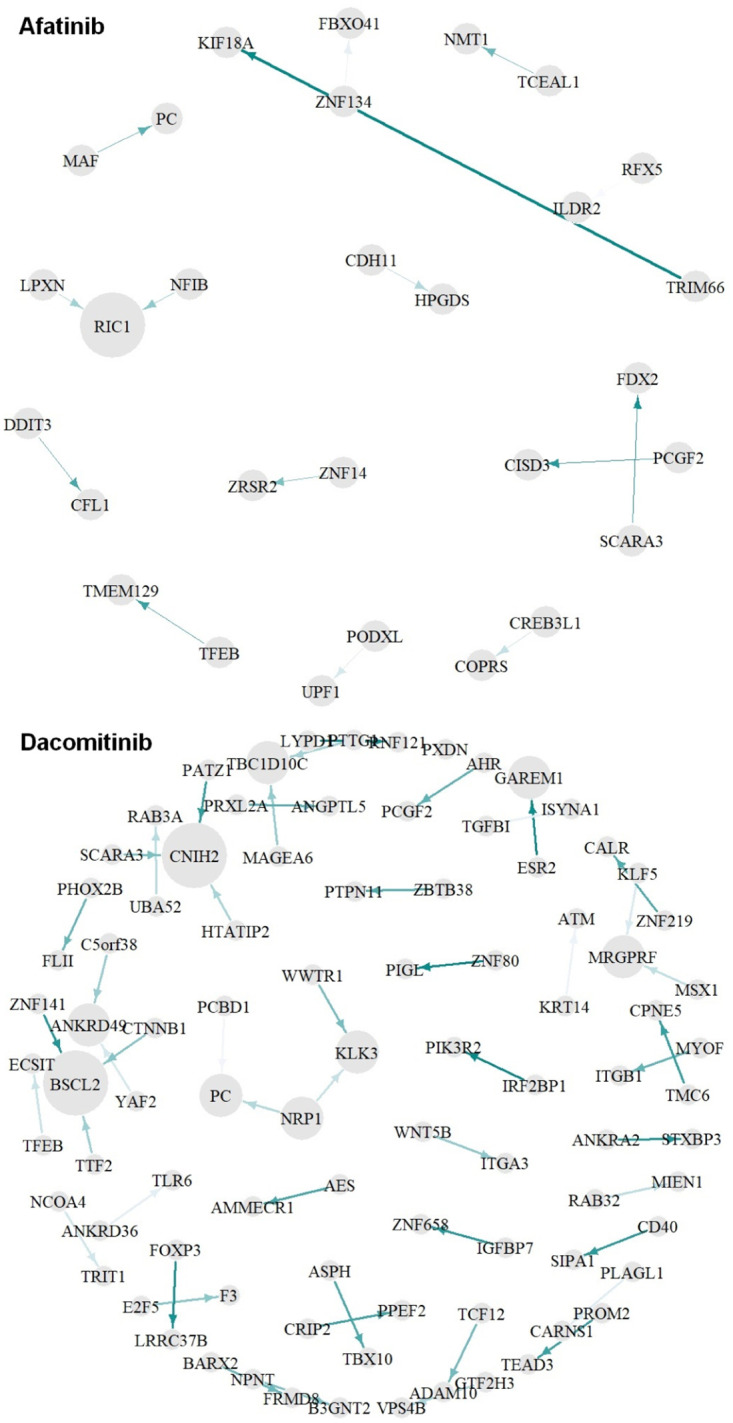
Crucial molecular interaction for afatinib/dacomitinib sensitivity prediction.

For the best models of each drug, we computed prediction accuracies based on the network without the (*l*, *j*)^*th*^ interaction $\text{Acc}\left({\hat{y}}^{\left(l,j\right)}\right)$, where the prediction accuracy is measured by 50 times repeated 10-fold cross validation processes. We then performed t-test between $\text{Acc}\left({\hat{y}}^{\left(l,j\right)}\right)$ and $\text{Acc}\left(\hat{y}\right)$ from the 50 interactions and computed the p.value of each gene-gene interaction. The edges having a p.value smaller than 0.01 are considered as crucial molecular interactions for drug sensitivity prediction. The overall framework of explainable EGFR TKIs prediction based on Xprediction is given in Fig 1. For the dacomitinib sensitivity prediction, many edges were extracted. In contrast, the least number of edges were identified for afatinib prediction (i.e., afatinib: 15, dacomitinib: 549, erlotinib: 249, gefitinib: 325, and osimertinib: 223 edges were extracted as a crucial feature). The distribution of importance of the edges is given in Fig 2. All crucial edges and their crucialness (i.e., p.values) can be found in the S2 Table. We visualize importance of gene-gene interaction for afatinib and dacomitinib sensitivity prediction. For dacomitinib, we extracted the crucial 50 edges (i.e., edges corresponding to the smallest 50 p.values) to visualize. Fig 3 shows the crucialness of the molecular interactions and their gene regulatory networks. Edge thickness and darkness of color represent the crucialness of each edge on prediction, where the crucialness was measured based on a p.value. Node size represents hubness (i.e., node sizes based on the degree of connectivity of the nodes) of each gene in the networks. As shown in Fig 3, Xprediction can describe importance of input features of machine/deep learning models; thus, we can interpret the prediction results based on crucial biomarkers. In other words, our methods provide effective drug sensitivity prediction accuracy compared with expression-based prediction and present interpretable results for drug sensitivity prediction. The networks of the crucial 50 edges for erlotinib, gefitinib and osimertinib sensitivity prediction are given as S1–S3 Figs, respectively.

**Fig 4:**
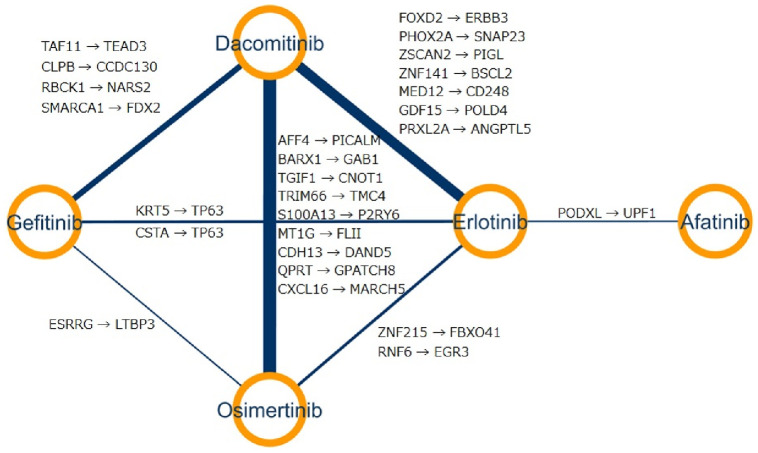
EGFR-TKI networks: Two drugs are connected if they have common crucial molecular interaction for prediction of their sensitivities, and thickness of edges indicates number of common crucial molecular interaction.

Table 2 shows the most crucial five molecular interactions corresponding to the smallest p.values. The column “PN” indicates positive (+) and negative (-) features for prediction accuracy, i.e., $+:\hat{y}-{\hat{y}}^{\left(l,j\right)}>0$ and $-:\hat{y}-{\hat{y}}^{\left(l,j\right)}<0$. Molecular interactions of TP63 (CSTA → TP63 and KRT5 → TP63) were identified as the most crucial feature to predict erlotinib sensitivity. Furthermore, the interactions of TP63 were extracted as a crucial feature for gefitinib sensitivity prediction (see Fig 4). It implies that TP63 and its interaction with CSTA and KRT5 can be considered candidate markers related to EGFR-TKIs sensitivity.

**Table 2:** The most crucial five molecular interactions for drug sensitive prediction.

Drug	Network	Regulator	Target	p.value	PN
afatinib	dacomitinib	TRIM66	KIF18A	0.002	+
		SCARA3	FDX2	0.004	-
		PCGF2	CISD3	0.004	-
		TFEB	TMEM129	0.005	+
		DDIT3	CFL1	0.006	+
dacomitinib	afatinib	HOXC4	UBAP1	0.000	-
		PROP1	CPNE5	0.000	-
		SUPT3H	DAND5	0.000	-
		MAFG	RAB35	0.000	-
		NOTCH3	ENDOU	0.000	-
erlotinib	afatinib	CSTA	TP63	0.000	+
		KRT5	TP63	0.000	+
		NR4A1	IRGC	0.000	+
		ZBTB17	RELT	0.000	+
		MDM2	ANGPTL5	0.001	+
gefitinib	osimertinib	ZIC1	NARS2	0.000	-
		RELB	LST1	0.000	-
		NFX1	RIC1	0.000	-
		PSMC3	UBA6	0.000	-
		FOXO3	IRF2BP2	0.000	-
osimertinib	afatinib	SFRP1	MRGPRF	0.000	+
		S100A13	P2RY6	0.000	+
		EID1	ST20	0.001	+
		GLI1	TSPAN16	0.001	+
		HOXD11	BCAS2	0.001	+

A majority of the identified genes in including TP63 and its interactions have been verified as biomarkers for prognosis and diagnosis of NSCLC. Their mechanism related to EGFR-TKIs sensitivity is also revealed as follows.

- Markers for prognosis and diagnosis of NSCLC
  Variation in TP63 is associated with lung-adenocarcinoma (AC) susceptibility in Japanese and Korean populations [16]. It was revealed through gene enrichment analysis that TP63 was an important upstream regulator, with elevated expression in SCC compared to that in AC [17]. KRT5 and TP63 are known as the squamous cell carcinoma (SCC) of the lung-specific genes [18]. Exosomal TP63, KRT5, CEACAM6, and SFTPB mRNAs can be used as biomarkers to differentiate between lung squamous cell carcinoma (LUSC) and lung squamous cell carcinoma (LUAD), thereby, providing a novel strategy for their differential diagnosis and treatment [19]. KRT5 and TP63 were selected as the biomarkers for distinguishing AC from SCC (subtypes of NSCLC), as well as novel molecular targets for targeted therapeutic agents [20, 21]. CSTA is one of the target molecules of TP63, and its expression was in the same predicted direction as the TP63 activation in SCC. TP63 and CSTA play an important role in epithelial tissue maintenance and development, and they are markers of squamous differentiation [22]. TRIM66 is involved in the targetable axis for the treatment of NSCLC [23]. CSTA was identified as a squamous cell lung carcinoma-associated gene [24]. TP63 and TTF-1 are useful for distinguishing both small cell carcinomas as well as AC from SCC [25]. NR4A1 is a potential therapeutic target for non-smoking female NSCLC patients [26]. Overexpression of S100A13 protein is associated with tumor angiogenesis, and poor survival in patients with early-stage NSCLC [27]. The mRNA level of CFL1 in NSCLC can discriminate between good and bad prognosis, in which tumors with high expression of CFL1 are associated with low survival of NSCLC [28]. HOXD11 is an upregulated gene in AC and SCC and is mainly associated with extracellular matrix and proliferation, promotion of tumorigenesis, and apoptotic processes [29]. High RELB expression is a marker for judgment of prognosis in patients with NSCLC [30]. SFRP1 is a candidate for epigenetic therapy in NSCLC [31].
- Markers for EGFR-TKIs sensitivity
  TP63 was identified as a differentially expressed gene (DEG) in cancer cell lines that acquired gefitinib resistance, and CSTA is one of the top 10 hub genes in a network of DEGs for gefitinib resistant cancer cell line [32]. TP63 expression is associated with survival outcomes in patients with NSCLC, treated with erlotinib [33]. MDM2 is a novel biomarker and treatment target for NSCLC and confers primary resistance to first-generation EGFR-TKIs induced by MDM2 amplification in NSCLC [34]. The whole-exome sequencing of osimertinib-resistant patient-derived cancer cell lines revealed amplification of GLI1, CDK4, and CCND1 [35]. GLI1 activation is a key mechanism of erlotinib resistance in human NSCLC [36]. A study by Mambetsariev reported five mutations, CDK4 amplification, MDM2 amplification, FRS2 amplification, GLI1 amplification, and the EGFR exon 19 deletion,via a tissue biopsy test, a few weeks into the osimertinib treatment [37]. FOXO3a is a significant factor in EGFR mutation-independent gefitinib resistance. It suggests that targeting the NF-kB/miR-155/FOXO3a pathway has potential therapeutic value in lung cancer with the acquisition of resistance to EGFR-TKIs [38]. The transcription factor FOXO3a is a crucial cellular target of gefitinib in breast cancer cells [39]. FOXO3 polymorphisms were correlated with gefitinib-induced hepatotoxicity in patients NSCLC [40]. EGFR TKI therapy activates *β*-catenin signaling in a NOTCH3 dependent manner. NOTCH3, *β*-catenin, and EGFR regulate each other, and EGFR TKI therapy mediated NOTCH3 activation leads to *β*-catenin activation, which is essential for the maintenance of drug persister cells in a positive feedback loop [41]. The treatment of EGFR-mutated lung cancer cell lines with erlotinib enriched then stem-like cells with stem-like cell potential through EGFR-dependent activation of NOTCH3 [42]. CircPSMC3 overexpression increased the sensitivity of esophageal SCC cells to gefitinib. It was suggested that upregulation of circPSMC3 overcame the resistance of gefitinib-resistant esophageal SCC cells to gefitinib by modulating the miR-10a-5p/PTEN axis, which provides a new therapeutic strategy for overcoming gefitinib resistance in esophageal SCC [43].

From these results, it can be considered that the identified crucial genes for EGFR-TKI prediction are prognostic and diagnostic markers of NSCLC. Furthermore, these genes have vital clues to uncover the molecular mechanisms related to EGFR-TKI sensitivity of cell lines. It implies that our method provides biologically reliable results for explainable drug sensitivity prediction.

### EGFR-TKIs network

We constructed EGFR-TKIs network based on the selected crucial edges in Fig 4, where two drugs are connected if they have at least one common edge.

The sensitivities of dacomitinib and osimertinib were explained by the most common molecular interaction, thereby, implying that dacomitinib and osimertinib have similar mechanisms of action and pharmacological profiles. Previous studies support our result that the combination of osimertinib and dacomitinib could induce more durable responses by preventing the emergence of resistance [44]. The sensitivity of almost EGFR-TKIs can be explained by common molecular interactions, while afatinib has common features with only erlotinib.

**Fig 5:**
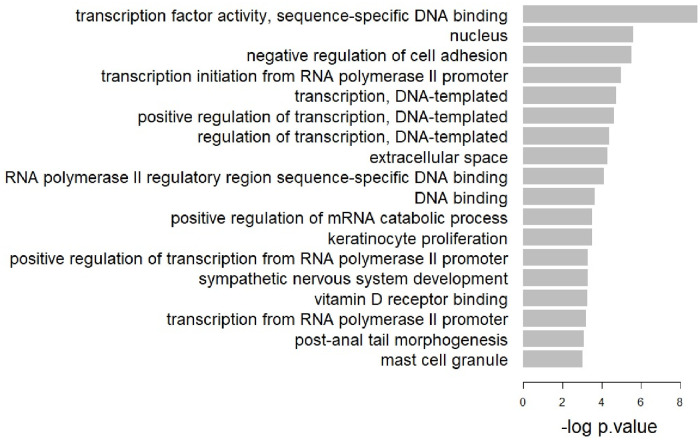
GO enrichment analysis of the common markers for EGFR TKIs.

To identify the biological process, molecular function, and cellular component of the crucial markers for predicting EGFR TKIs, we perform Gene Ontology (GO) pathway analysis. Fig 5 shows the enriched pathway (*p* <.01) of the genes consisting of the common edges and corresponding p-value (i.e., −log(p.value)). GO enrichment analysis revealed that the 51 EGFR TKIs markers are enriched in pathways involving *transcription factor activity, transcription initiation from RNA polymerase II promoter, transcription, positive regulation of transcription, regulation of transcription*, etc. This implies that the EGFR TKIs markers may dominate EGFR TKIs sensitivity-specific gene networks by transcription.

### EGFR-TKIs sensitive and resistant specific molecular networks system

**Fig 6:**
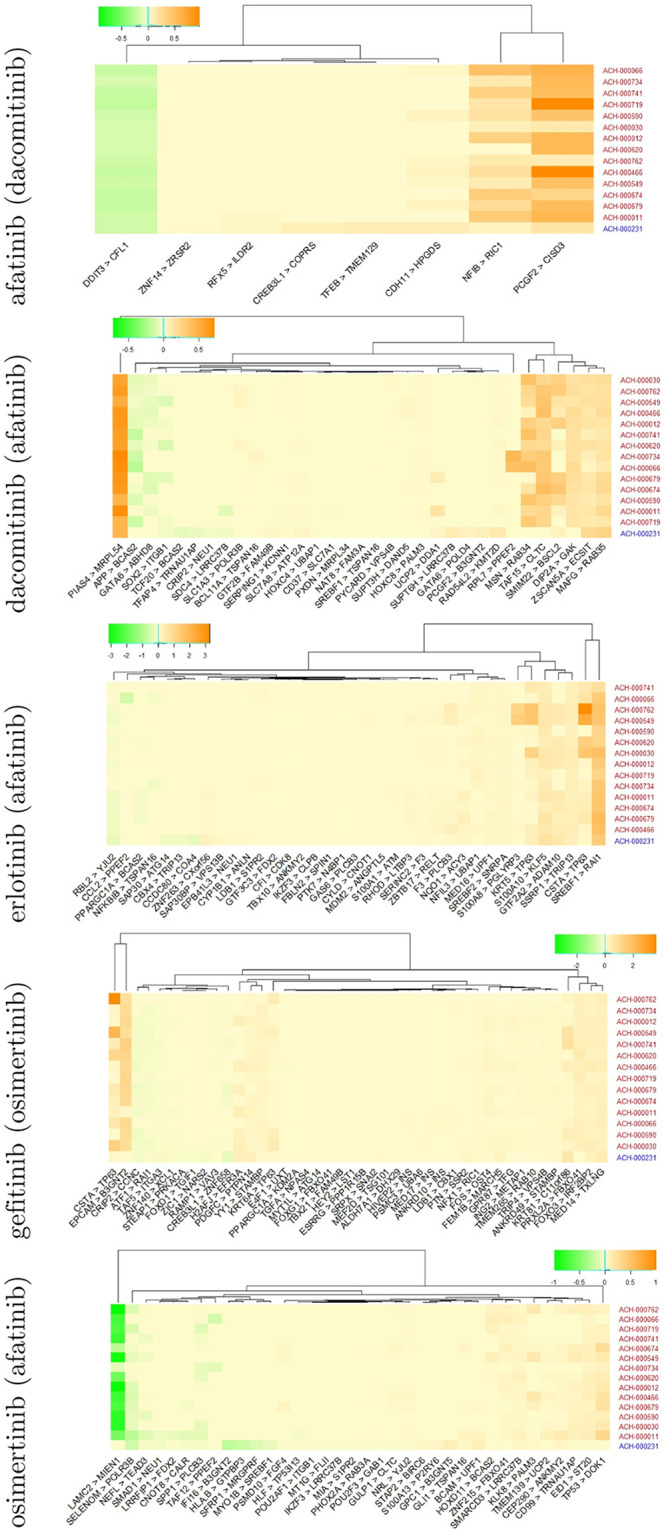
Regulatory effect of crucial molecular interaction on the commonly sensitive and resistant cells of the EGFR TKIs, where color of cell lines (rows) indicates drug sensitive (red) and resistant (blue) cells.

We focused on common sensitive (ST) and resistant (RS) cells of the five EGFR TKIs. Table 3 shows information of the cell lines, where ACH-000011 is a cell line sensitive to afatinib, dacomitinib, erlotinib, gefitinib, and osimertinib, while ACH-000231 is a cell line resistant to the five EGFR TKIs. The EGFR TKIs sensitive cell lines include non-small cell lung cancer (NSCLC), bladder carcinoma, and upper aerodigestive squamous cells. For the sensitive and resistant cell lines of the EGFR TKIs, we showed the regulatory effect of the most crucial 50 edges described in Fig 3. Fig 6 shows regulatory effect values of the regulator on the target gene (column indicates regulator > target) for common sensitive and resistant cell lines of the EGFR TKIs, wherein the color of cell lines in rows of the heatmap is used to indicate sensitive (red) and resistant (blue) cell line of EGFR TKIs. Some edges showed different regulatory systems between EGFR TKIs resistant and sensitive cell lines, suggesting that some edges exist in only EGFR TKIs resistant or sensitive cell lines. CREB3L1>COPRS and ZNF14>ZRSR2 can be considered afatinib resistant specific molecular interaction. For dacomitinib, APP>BCAS2, RPL7>PPEF2, MSN>RAB34, TAF15>CLTC and DIP2A>GAK show large regulatory effect values in sensitive cell lines, while TCF20>BCAS2, SUPT6H>LRRC37B, GATA6>POLD4 and PCGF2>B3GNT2 can be considered resistant markers. SAP30BP>VPS13B, CBX4>TRIP13 and CCDC80>COA4 are considered as erlotinib resistant specific markers. CSTA>TP63 and EPCAM>B3GNT2 show relatively large regulatory effect in gefitinib sensitive cell lines than its resistant cells. For osimertinib, LRRFIP1>FDX2 is considered as drug sensitive specific molecular interaction, and FI16>B3GNT2, HLA.B>GTPBP3, SFRP1>MRGPRF and MYO1B>SREBF1 show large regulatory effect values in resistant cell lines. Table 4 shows drug sensitive and resistant specific molecular markers of each EGFR TKIs, where sensitive and resistant markers are interactions existed only in drug sensitive and resistant cell lines, respectively.

**Table 3:** Information of cell lines.

DepMap ID	Drug sensitivity	sex	age	primary disease	lineage subtype
ACH-000011	ST	Male	53	Bladder Cancer	bladder carcinoma
ACH-000012	ST	Female	39	Lung Cancer	NSCLC
ACH-000030	ST	Male	NA	Lung Cancer	NSCLC
ACH-000066	ST	Male	NA	Lung Cancer	NSCLC
ACH-000466	ST	Female	46	Gastric Cancer	gastric adenocarcinoma
ACH-000549	ST	Male	60	Head and Neck Cancer	upper aerodigestive squamous
ACH-000590	ST	Female	47	Lung Cancer	NSCLC
ACH-000620	ST	Male	50	Liver Cancer	hepatocellular carcinoma
ACH-000741	ST	Female	NA	Bladder Cancer	bladder carcinoma
ACH-000674	ST	Female	35	Gastric Cancer	gastric adenocarcinoma
ACH-000679	ST	Male	72	Esophageal Cancer	esophagus squamous
ACH-000719	ST	Female	34	Ovarian Cancer	ovary adenocarcinoma
ACH-000734	ST	Male	50	Liver Cancer	hepatocellular carcinoma
ACH-000762	ST	Male	67	Head and Neck Cancer	upper aerodigestive squamous
ACH-000231	RS	Female	NA	Brain Cancer	glioma

**Table 4:** Drug sensitivity specific markers.

	afatinib	dacomitinib	erlotinib	gefitinib	osimertinib
Resistant markers	ZNF14>ZRSR2	TFAP4>TRNAU1AP	TBX10>ANKMY2	MEF2D>TSG101	SFRP1>MRGPRF
	CREB3L1>COPRS	SUPT6H>LRRC37B	CBX4>TRIP13	FOXD1>TGIF1	S100A13>P2RY6
		PCGF2>B3GNT2	CCDC80>COA4	SRPX>SNAI2	HLA.B>GTPBP3
		CRIP2>NEU1	SAP30BP>VPS13B		IFI16>B3GNT2
		TCF20>BCAS2	EPB41L3>NEU1		GPC1>B3GNT5
		PXDN>MRPL34	CYP1B1>ANLN		STAP2>BIRC6
		GTF2B>FAM49B	SAP30>ATG14		MYO1B>SREBF1
		GATA6>POLD4			MT1G>FLII
Sensitive markers		HOXC4>UBAP1	ZBTB17>RELT	PSMC3>UBA6	EID1>ST20
		SUPT3H>DAND5	MDM2>ANGPTL5	LDB1>CBX1	HOXD11>BCAS2
		SLC1A3>POLR3B	LDB1>S1PR2	FOS>OST4	LRRFIP1>FDX2
		DIP2A>GAK	CFI>CDK8	TRIP4>VPS4B	ZNF215>FBXO41
		TAF15>CLTC	CYLD>CNOT1	EPCAM>B3GNT2	PHOX2A>RAB3A
		APP>BCAS2	NFIL3>UBAP1	ALDH7A1>DHX29	TAF12>PPEF2
		BCL11A>TSPAN16	IKZF3>CLPB	YY1>STAMBP	POU2F3>GAB1
		RPL7>PPEF2	GTF3C3>FDX2	MED17>INS	CD99>TRNAU1AP
		PYCARD>VPS4B	FBLN2>SPIN1	ESRRG>PPP1R15B	NRL>YJU2
		HOXC8>PALM3	S100A1>ATM	ANKRD10>INS	TMEM139>UCP2
		GATA6>ABHD8	PTK7>N4BP1	GPR87>TFG	TP53>DOK1
		NAT8>FAM3A	CCL2>PPEF2	TGFA>NPAS4	LAMC2>MIEN1
		CD37>SLC7A1	NFKBIB>TSPAN16	MYT1L>ATG14	POU2AF1>ITGB1
		MSN>RAB34	GAS6>PLCB3	PRXL2A>FBXO41	MIA>S1PR2
		SREBF1>TSPAN16		TMEM265>RAB10	PSMD10>FGF4
				HEY2>LST1	GULP1>CLTC
				HIVEP2>INS	
				ING2>METAP1	
				E4F1>UXT	
				PPARGC1A>KDM2A	
				TBX21>FAM49B	
				FOXG1>FBXO41	

**Fig 7:**
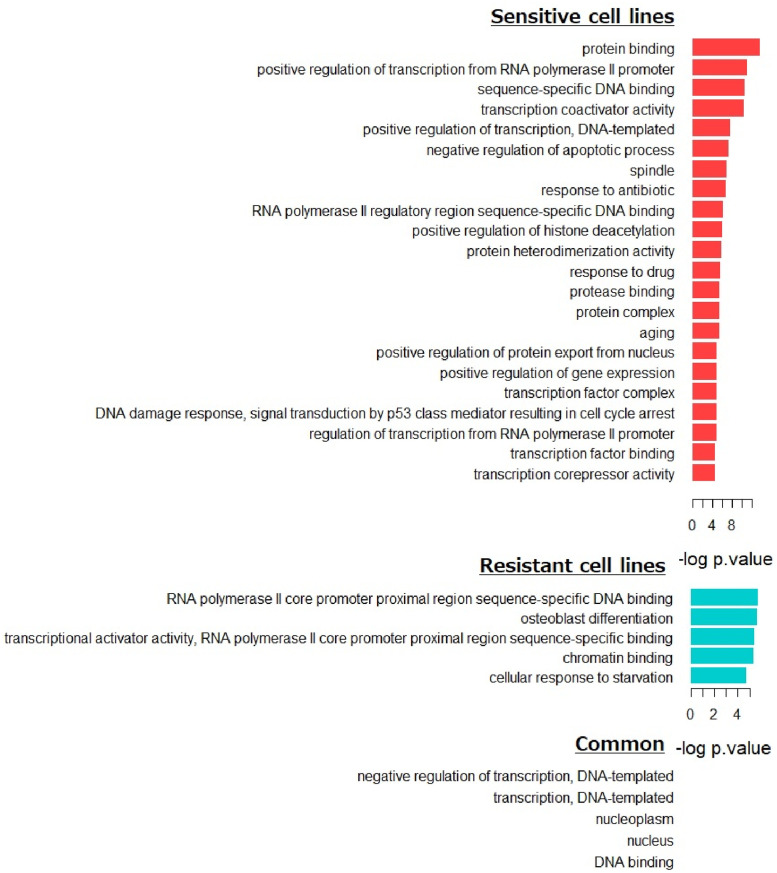
GO enrichment analysis for the sensitive and resistant markers.

We performed GO pathway analysis of the drug sensitive and resistant specific markers. Fig 7 shows the GO pathway of the identified markers, where colors are used to indicate enriched pathways for drug sensitive (red) and resistant (blue) specific markers. The bottom part of the Fig 7 shows the enriched pathways for both sensitive and resistant markers. As the pathways have different signs on the sensitive and resistant specific cell lines (i.e., −log(p.value)), we do not describe the significance. As shown in Fig 7, drug sensitive-specific markers were enriched in pathways involving *protein binding, positive regulation of transcription from RNA polymerase II promoter, sequence-specific DNA binding*, etc. Meanwhile, pathways involving *RNA polymerase II core promoter proximal region sequence-specific DNA binding, osteoblast differentiation, transcriptional activator activity, RNA polymerase II core promoter proximal region sequence-specific binding*, were identified for drug resistant specific markers. Interestingly, *response to drug* (GO:0042493) and *response to antibiotic* (GO:0046677) were identified as pathways for drug sensitive specific markers, where SREBF1, NAT8, MDM2, GATA6, SLC1A3, TGFA, FOS, PPARGC1A were enriched in the pathway *response to drug* and MDM2, SLC1A3, CCL2, TP53 were enriched in pathway *response to antibiotic*.

The results of Figs 6 and 7, Table 4, indicate that EGFR TKIs -resistant and -sensitive cell lines have different molecular regulatory systems. The drug sensitive/resistant specific gene regulatory system may be crucial clues to predict drug sensitivity and uncover molecular mechanisms underlying the drug sensitivity of cell lines.

## Discussion

We introduced a novel strategy for explainable drug sensitivity prediction based on sample-specific gene regulatory networks. We focused not only on drug sensitivity prediction but also on the interpretation of the prediction results. To understand complex disease mechanisms and effectively predict drug response of cancer cell lines, we considered drug response prediction based on gene regulatory networks under varying conditions of cell lines. To overcome the black box problem of machine learning approaches in drug sensitivity prediction (i.e., interpretability of the prediction results), we proposed a method that reveals the importance of molecular interaction on the results of prediction model based on machine/deep learning approaches. We can explain the drug sensitivity prediction results and perform interpretable drug sensitivity prediction by using the proposed method.

To illustrate our strategy, we applied the proposed Xprediction to EGFR-TKIs prediction. It can be seen that the drug sensitivity-specific gene network-based prediction provides an effective result for EGFR-TKIs prediction, compared with the prediction based on expression levels of genes. We identified important markers for EGFR-TKIs prediction, and mechanisms related EGFR-TKIs of the majority of the identified markers are verified based on literature. It implies that genetic networks can predict crucial information and understand the drug sensitivity of cancer cell lines, which cannot be extracted by single gene-based analysis.

In this study, we focused on five EGFR-TKIs, and predicted the sensitivity of a EGFR-TKI based on other EGFR-TKIs sensitivity specific networks. Our strategy can be extended to anti-cancer drug response prediction based on various cancer characteristic specific gene networks, e.g., status of caner progress, survival risk and clinical feature -specific gene networks.

Further work remains to be done towards experimental validation of the identified markers to provide strong evidence of our results. In this study, we performed drug sensitivity prediction based on estimated sample-specific gene networks. In other words, explainable drug sensitivity prediction was performed by two separate models for gene network estimation and drug response prediction. To achieve more effective prediction and interpretable results of drug sensitivity prediction, we shall consider a model that simultaneously performs drug response prediction and gene network estimation as one of our future works.

## Supporting information

S1 Fig The networks of the crucial 50 edges for erlotinib, gefitinib and osimertinib sensitivity prediction. (JPG)

S2 Fig The networks of the crucial 50 edges for erlotinib, gefitinib and osimertinib sensitivity prediction. (JPG)

S3 Fig The networks of the crucial 50 edges for erlotinib, gefitinib and osimertinib sensitivity prediction. (JPG)

S1 Table All pan-cancer interactions extracted from five EGFR TKIs sensitivity specific gene networks. (XLSX)

S2 Table All crucial edges and their crucialness (i.e., p.values). (XLSX)
