# Supplementary figures and images for: Xprediction: Explainable EGFR-TKIs response prediction based on drug sensitivity specific gene networks
Source: PLoS One. 2022 May 18;17(5):e0261630. doi: 10.1371/journal.pone.0261630 (PMC9116684; doi:10.1371/journal.pone.0261630)

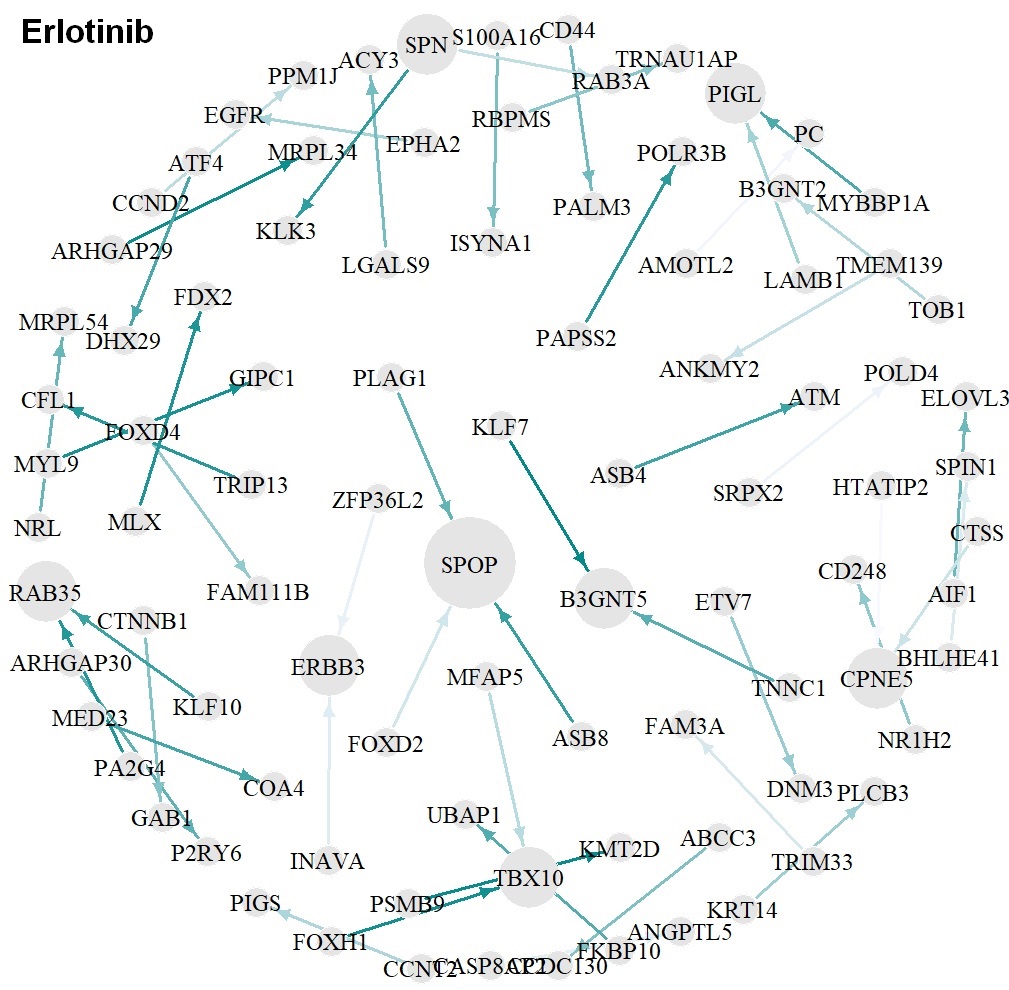

Supplement: S1 Fig — (JPG) [file pone.0261630.s001.jpg]

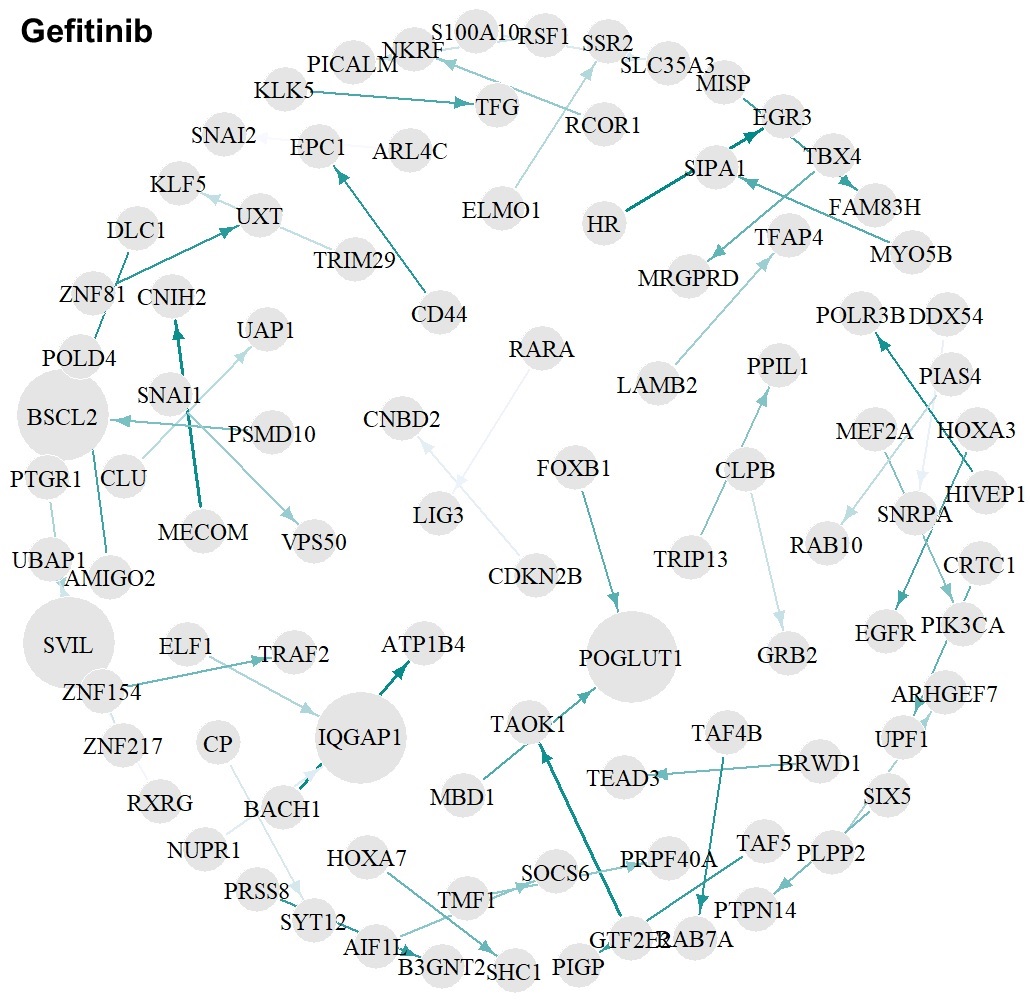

Supplement: S2 Fig — (JPG) [file pone.0261630.s002.jpg]

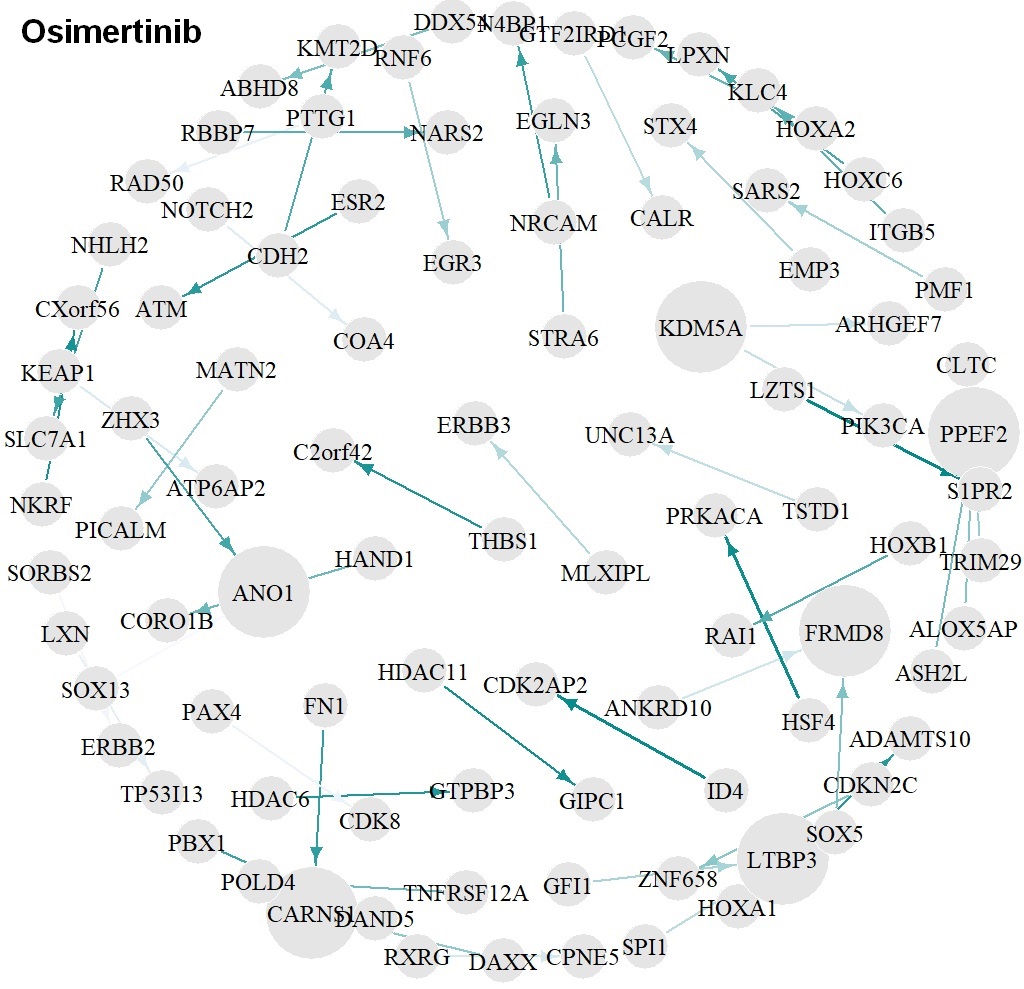

Supplement: S3 Fig — (JPG) [file pone.0261630.s003.jpg]
